# Optimised GMP-compliant production of [^18^F]DPA-714 on the Trasis AllinOne module

**DOI:** 10.1186/s41181-021-00133-0

**Published:** 2021-05-26

**Authors:** Klaudia A. Cybulska, Vera Bloemers, Lars R. Perk, Peter Laverman

**Affiliations:** 1 Radboud Translational Medicine B.V., Geert Grooteplein 21, Nijmegen, 6525 EZ Netherlands; 2 Department of Radiology and Nuclear Medicine, Radboud University Medical Center, Geert Grooteplein 10, Nijmegen, 6525 GA Netherlands

**Keywords:** [^18^F]DPA-714, TSPO, PBR, PET, Fluorine-18, Radiochemistry, Radiopharmacy, GMP, Trasis AllinOne, Neuroinflammation

## Abstract

**Background:**

The translocator protein 18 kDa is recognised as an important biomarker for neuroinflammation due to its soaring expression in microglia. This process is common for various neurological disorders. DPA-714 is a potent TSPO-specific ligand which found its use in Positron Emission Tomography following substitution of fluorine-19 with fluorine-18, a positron-emitting radionuclide. [^18^F]DPA-714 enables visualisation of inflammatory processes in vivo non-invasively. Radiolabelling of this tracer is well described in literature, including validation for clinical use. Here, we report significant enhancements to the process which resulted in the design of a fully GMP-compliant robust synthesis of [^18^F]DPA-714 on a popular cassette-based system, Trasis AllinOne, boosting reliability, throughput, and introducing a significant degree of simplicity.

**Results:**

[^18^F]DPA-714 was synthesised using the classic nucleophilic aliphatic substitution on a good leaving group, tosylate, with [^18^F]fluoride using tetraethylammonium bicarbonate in acetonitrile at 100^∘^C. The process was fully automated on a Trasis AllinOne synthesiser using an in-house designed cassette and sequence. With a relatively small precursor load of 4 mg, [^18^F]DPA-714 was obtained with consistently high radiochemical yields of 55-71% (n=6) and molar activities of 117-350 GBq/µmol at end of synthesis. With a single production batch, starting with 31-42 GBq of [^18^F]fluoride, between 13-20 GBq of the tracer can be produced, enabling multi-centre studies.

**Conclusion:**

To the best of our knowledge, the process presented herein is the most efficient [^18^F]DPA-714 synthesis, with advantageous GMP compliance. The use of a Trasis AllinOne synthesiser increases reliability and allows rapid training of production staff.

## Introduction

The translocator protein (18 kDa), TSPO in short, is found on the outer mitochondrial membrane and has the highest expression in steroidogenic tissues (Lee et al. 2020; Choi et al. 2011). The protein was formerly known as the peripheral benzodiazepine receptor (PBR), the name, however, was judged to misrepresent its role, following extensive characterisation. Neither were benzodiazepines its only binding targets (omitting cholesterol, among others), nor was "peripheral" an accurate description of its localisation, as one its expression sites is the brain itself (Papadopoulos et al. 2006). Typically, TSPO expression in the central nervous system (CNS) is modest and limited primarily to glial cells, particularly astrocytes and microglia (Selvaraj and Stocco 2015). A marked increase in TSPO density is observed as a result of neuroinflammation mediated by the activation of microglia. This mechanism is implicated in various neuropathologies and is the common denominator in their early stages. TSPO expression has been suggested as a biomarker for brain disorders characterised by activated microglia, such as Alzheimer’s disease (AD), multiple sclerosis (MS), stroke and cancer (James et al. 2008).

Positron Emission Tomography (PET) is a powerful molecular imaging technique for detection of TSPO density changes, with the potential to become a useful diagnostic tool for neuroinflammatory responses, particularly relevant in early disease stages. The first TSPO targeting tracer, (*R*)-[^11^C]PK11195 (1-(2-chlorophenyl)-*N*-[^11^C]methyl-*N*-(1-methylpropyl)-3-isoquinoline carboxamide), was employed in several studies to investigate microglial activation, however its suboptimal non-specific binding component deteriorates image quantification due to the low signal-to-noise ratio (Guo et al. 2012). Extensive research led to the elucidation of numerous ligands, including [^11^C]PBR28 (*N*-acetyl-*N*-(2-[^11^C]methoxybenzyl)-2-phenoxy-5-pyridinamine) and [^18^F]PBR111 ((2-(6-chloro-2-(4-(3-[^18^F]fluoropropoxy)phenyl)imidazo[1,2-*a*]pyridin-3-yl)-*N,N*-diethylacetamide), known collectively as second-generation TSPO tracers. The former exhibited particularly favourable kinetics and specificity for the protein of interest (Collste et al. 2016). A clear advantage of [^18^F]PBR111 over [^11^C]PBR28 is the use of fluorine-18, exhibiting superior decay properties and consequently, higher resolution images. In addition, similar improvements with respect to the signal-to-noise ratio of the PET images were reported (Eberl et al. 2017). Recently, a structural derivative of [^18^F]PBR111, [^18^F]DPA-714 or (*N,N*-diethyl-2-(2-(4-(2-[^18^F]fluoroethoxy)phenyl)-5,7-dimethylpyrazolo[1,5-*a*]pyrimidin-3-yl)acetamide received significant attention as a superior TSPO ligand, owing to high specific binding and brain-blood barrier penetration (Golla et al. 2015). Biodistribution and blocking studies with unlabelled DPA-714 and PK11195 were performed by Vicidomini and co-workers using µPET in the brain and peripheral tissues of mice (Vicidomini et al. 2015). Golla et al. employed the tracer in a study with 10 AD patients and 6 healthy volunteers to determine the appropriate kinetic model for data quantification (Golla et al. 2015). Recently, Hagens et al. performed a proof-of-concept study with 8 MS patients and 7 healthy controls to evaluate the imaging power of the tracer in progressive MS (Hagens et al. 2018). Promising results of various investigations have fuelled efforts into optimising radiosynthesis of [^18^F]DPA-714 for clinical use, requiring a robust and high-yielding process, with an appropriate degree of automation for multi-patient and multi-centre clinical trials.

Initial synthetic efforts were described by James et al., who achieved ^18^F-labelling of the tosylate DPA-714 precursor via S_N_2 substitution with [^18^F]fluoride in the presence of Kryptofix-222 and potassium carbonate in refluxing acetonitrile for 10 min. The production was performed on a GE TRACERlab MX_FDG_ system. Decay-corrected (dc.) radiochemical yields (RCYs) were modest, reaching 21% only with a high precursor loading of 6-12 mg (James et al. 2008). Damont and co-workers performed the radiolabelling analogously, however, exchanging acetonitrile for DMSO at 165^∘^C and shortening the reaction time to 5 min (Damont et al. 2008). Pleasingly, this led to an increase of RCY (ndc.) up to 20%, corresponding to 35% dc., with molar activities (A_m_) of 37-111 GBq/µmol at the end of synthesis (EOS). Kuhnast et al. published a detailed protocol of their high-yielding radiolabelling of the tracer on a TRACERlab FX_FN_ synthesiser (Kuhnast et al. 2012). The authors performed the reaction with tosylate DPA-714 in the presence of Kryptofix-222 and potassium carbonate to effectuate substitution of the tosylate moiety with [^18^F]fluoride. The transformation was achieved in either DMSO at 165^∘^C or MeCN at 120^∘^C, for 5 min in both circumstances, however the authors obtained significantly higher RCYs of 43-50% (dc.) with the high-boiling solvent. High A_m_ values were also obtained, reaching 222 GBq/µmol at EOS. Kuhnast et al. successfully led their [^18^F]DPA-714 synthesis through the GMP validation process, a critical step for clinical studies and potential marketing authorisation.

Herein, we describe efforts towards development of a robust [^18^F]DPA-714 radiosynthesis for clinical use at Radboud University Medical Center (Radboudumc), following a short yet fruitful optimisation, that resulted in, to the best of our knowledge, the most efficient and highest-yielding radiolabelling of the tracer reported in literature. Our fully GMP-compliant process was designed in-house on a Trasis AllinOne (AIO) synthesiser, making use of its simplicity, robustness and versatility, and in addition, enabling rapid training of radiopharmacy personnel.

## Materials and methods

### Materials and reagents

All reagents and solvents, unless specified otherwise, were purchased from Sigma Aldrich (Zwijndrecht, Netherlands) and VWR International (Amsterdam, Netherlands), and used without further purification. All materials were purchased from VWR International (Amsterdam, Netherlands), unless specified otherwise. Saline solution for injection (0.9% NaCl *w/v%*) and water for injection were acquired from B. Braun (Melsunger, Germany). The cassette was prepared in-house, starting from a commercially available [^18^F]FDOPA cassette from Trasis (Ans, Belgium). The GMP-grade precursor to [^18^F]DPA-714, DPA-714 tosylate, was purchased from Pharmasynth (Tallinn, Estonia). Sep-Pak Accell Plus QMA carbonate and Sep-Pak C18 Plus cartridges were purchased from Waters Corporation (Etten-Leur, Netherlands). Fluorine-18 for labelling was obtained on-site using a Siemens Eclipse HP cyclotron, in the form of [^18^O]H_2_O-bound [^18^F]fluoride via a ^18^O(p,n)^18^F nuclear reaction in a tantalum target. ^18^O-enriched water (≥97%) was purchased from Rotem Industries (Dimona, Israel). Cyclotron-specific parts are described in “Cyclotron” section.

### Optimisation of radiochemistry: manual labelling

Manual labelling was performed in a shielded fumehood from Von Gahlen (Zevenaar, Netherlands) with <1 GBq of cyclotron-produced [^18^F]fluoride (described extensively in “Cyclotron” section). Approximately 100-200 MBq of activity was used per reaction. Typically, [^18^F]fluoride in ^18^O-enriched water was passed through a Sep-Pak Accell Plus QMA cartridge. [^18^F]fluoride ions were eluted into a 5 mL V-shaped borosilicate reactor vial with a phase-transfer catalyst (PTC)/base solution of choice in a mixture of acetonitrile and water (Table 1). Subsequently, the reactor was heated to 90^∘^C to effectuate azeotropic distillation supported by a protective atmosphere of nitrogen gas. This was then repeated twice more with anhydrous acetonitrile (2 ×0.5 mL) at 90^∘^C until a dry off-white [^18^F]fluoride residue was obtained. The reactor was then cooled for 2 min before addition of the tosylate DPA-714 precursor solution (4.0 mg, 7.3 mmol) in anhydrous acetonitrile (1 mL). The reactor was sealed and the resulting mixture was heated at 90^∘^C for 10 min without stirring. The reactor vial was removed from the heat source and cooled for 2 min before addition of water (1.5 mL). A clear yellowish solution was obtained, of which an aliquot (20 µL) was analysed by HPLC (high performance liquid chromatography) coupled to a radioactivity detector (method described fully in “HPLC for identification, chemical and radiochemical purity determination” section).

**Table 1:** Results of the optimisation study with tosylate DPA-714 precursor in anhydrous acetonitrile at 90^∘^C after 10 min with different PTC/base systems. Each run was performed with the indicated volume of the PTC/base combination dissolved in acetonitrile/water 70/30 *v/v%* for Entry 1 and 85/15 *v/v%* for Entries 2 and 3

PTC	Base	Elution volume [mL]	Radiochemical yield [%]^*^	[^18^F]fluoride [%]^a^
K_222_ (37.2 mM)	K_2_CO_3_ (11.0 mM)	1	77	23
K_222_ (30.0 mM)	KHCO_3_ (30.0 mM)	0.75	84	15
TEAB (40.0 mM)	-	0.6	94	5

^a^non-isolated, estimated by (radio)HPLC

### Automated production

#### Trasis allinOne synthesiser

Radiosynthesis of [^18^F]DPA-714 was performed on a Trasis AllinOne (AIO) module (Ans, Belgium). The synthesiser is equipped with 36 rotary actuators (arranged in two rows) to which a dedicated disposable cassette can be attached. The cassette for [^18^F]DPA-714 productions was assembled in-house, starting from a commercially available Trasis [^18^F]FDOPA cassette, as described in “Cassette preparation” section.

#### Cyclotron

[^18^F]fluoride is produced using a Siemens Eclipse HP (11 MeV, 2 x 80 µA) cyclotron using 2-3 mL ^18^O-enriched water (>98%) contained in a tantalum target with a 50 µm thick Havar window. Typically, irradiation is performed with 60 µA 11 MeV protons for 20 min, yielding 30-40 GBq of [^18^F]fluoride at the end of bombardment (EOB). Transport of [^18^F]fluoride in [^18^O]H_2_O from the cyclotron target to the designated hot cell, through 35-40 m long 1/16” OD PTFE tubing from Bohlender GmbH (Grüsfeld, Germany), is effectuated using argon and helium gas (Argon Scientific 6.0, Helium Scientific 6.0, both from Linde Gas, Schiedam, Netherlands) in a two-stage delivery system. To ensure high A_m_ and RCYs, transfer lines towards the designated hot cell are rinsed with ^18^O-enriched water using an in-house rinsing system on the day of the production (preferably shortly before irradiation).

#### Cassette preparation

**Fig. 1:**
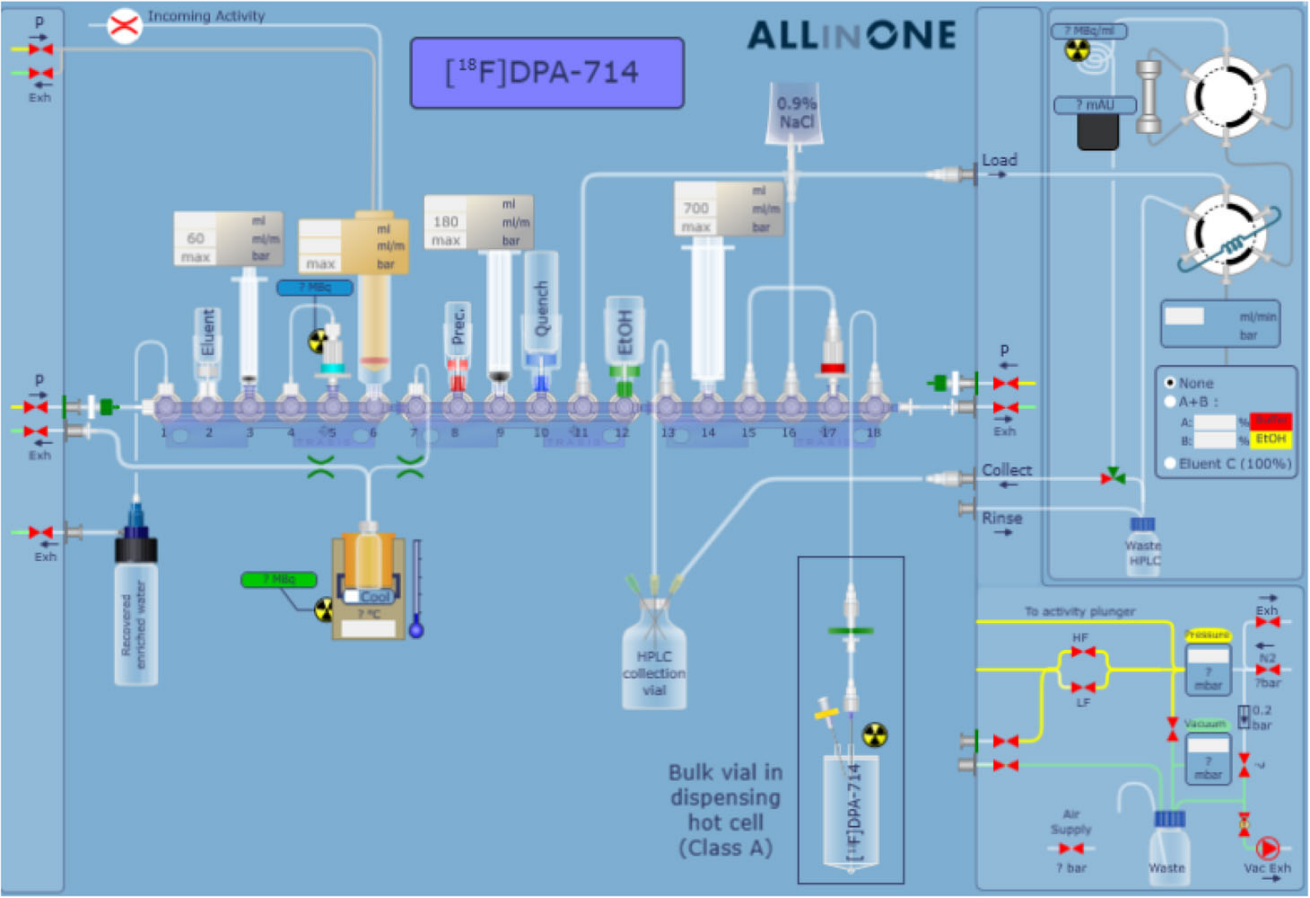
Layout of the cassette designed for the radiosynthesis of [^18^F]DPA-714 on a Trasis AIO module. The process can be controlled via the user software interface

**Fig. 2:**
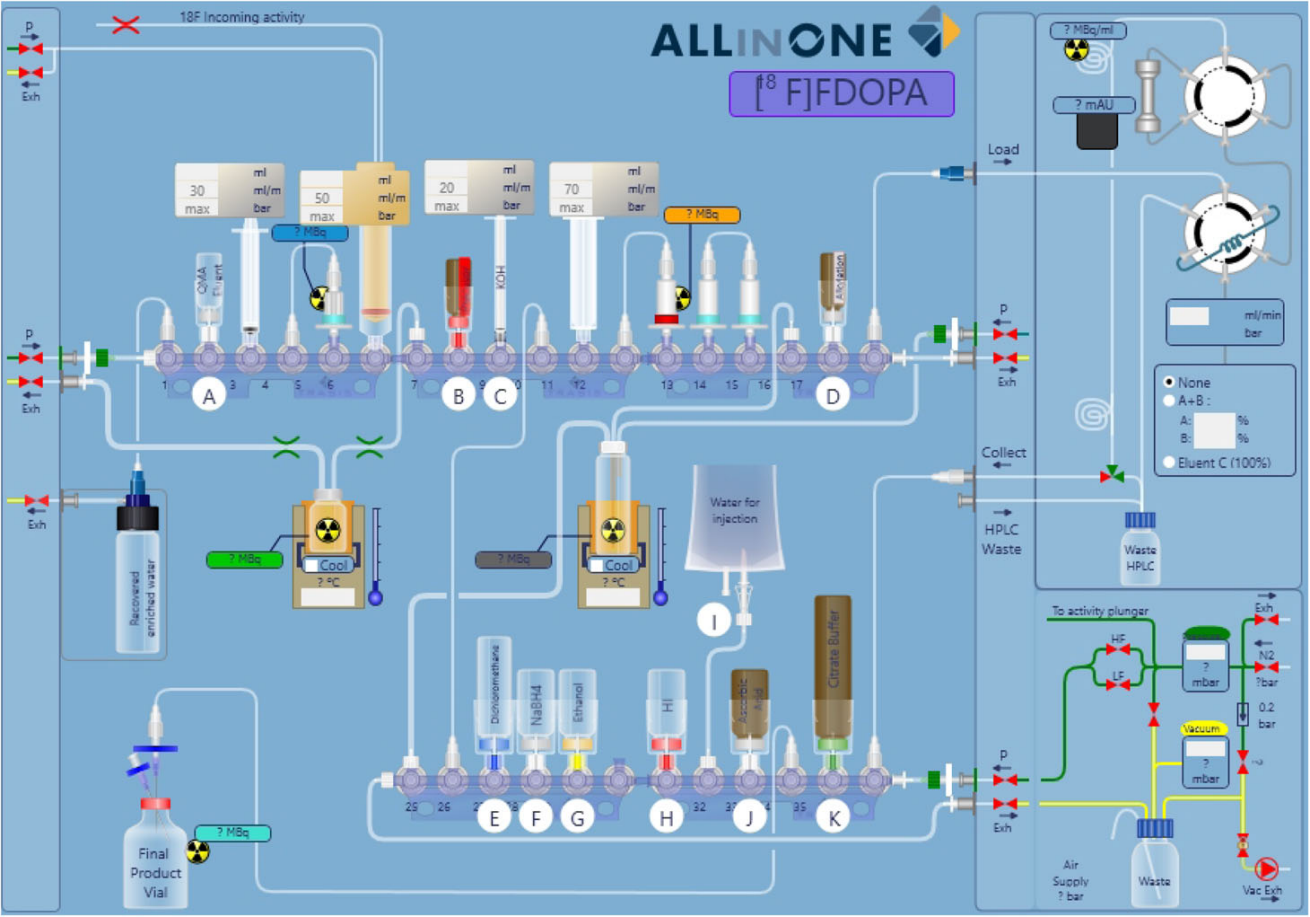
Layout of the commercially available cassette designed for the radiosynthesis of [^18^F]FDOPA on a Trasis AIO module by Trasis

The sequence and the corresponding cassette were developed in-house (Fig. 1). The [^18^F]FDOPA cassette served as a base for building the novel [^18^F]DPA-714 cassette, as it contained all the necessary components and required no additional purchases (Fig. 2).

All manipulations described herein were performed in a GMP Grade C environment. The cassette was removed from its wrapping and assembled no earlier than 24 hours prior to the start of synthesis (SOS). The [^18^F]DPA-714 process made use of valves 1-18 of the upper row of the synthesiser. Valves 1-9 and 18 of the original [^18^F]FDOPA cassette were left intact - transfer of [^18^F]fluoride from the cyclotron, trapping on a QMA ion-exchange cartridge, elution into the reactor, azeotropic distillation (1-6) and final product delivery (18) were the common denominator of both processes. All components attached to valves 10-17 were removed and recycled to build the desired cassette, according to the description in Table 2.

**Table 2:** Outline of the [^18^F]DPA-714 cassette on the Trasis AllinOne radiosynthesiser, with description of reagent and materials position. Positions outlined in boldface are original parts of the commercially available [^18^F]FDOPA cassette

**Manifold position**	**Reagents or materials attached**
**1 vertical**	PE tubing to [^18^O]H_2_O recovery vial
**1 horizontal**	Silicone tubing to pressure inlet
**2**	Spike, white Luer Lock ring and barrel for 20 mm vial
**3**	3 mL sterile syringe with Luer Lock
**4**	Silicone tubing connected to QMA cartidge in position 5
**5**	Activated QMA cartridge
**6**	3 mL sterile syringe with Luer Lock (activity inlet)
**7**	Reactor 6 mL with 2 tubes (male connector to cassette, female to exhaust)
**8**	Spike, red Luer Lock ring with barrel for 20 mm vial
9	10 mL BD PlastiPak sterile syringe with Luer Lock
10	Spike, blue Luer Lock ring
11	PE tubing OD 2 mm length 150 mm, connected to Load port of HPLC unit
12	Spike, green Luer Lock ring with barrel for 20 mm vial
13	PE tubing OD 2 mm length 150 mm, connected to 25 mL HPLC product receiving vial via sterile needle
14	20 mL HSW Norm-Ject sterile syringe with Luer Lock
15	PE tubing OD 2 mm length 100 mm, connected to Sep-Pak C18 Plus cartridge in position 17
16	0.9% NaCl for injection bottle, connected to valve using PE tubing OD 2 mm length 150 mm with spike
17	Activated Sep-Pak C18 Plus cartridge
**18 vertical**	Tubing to dispensing hot cell
**18 horizontal**	Silicone tubing to exhaust
**Other components**	**Reagents or materials attached**
Collect port HPLC unit	PE tubing OD 2 mm length 150 mm, connected to 25 mL HPLC product receiving vial via sterile needle
HPLC product collection 25 mL vial	Connected to cassette via tubing in Load port and postion 13
QMA reagent for elution (10 mL vial)	Connected to position 2 via spike
Precursor solution (10 mL vial)	Connected to position 8 via spike
Quench solution (25 mL vial)	Connected to position 10 via spike
Ethanol solution (10 mL vial)	Connected to position 12 via spike
0.9% NaCl for injection solution (250 mL bottle)	Connected to position 16 via tubing and spike

#### Synthesis preparation

**Preparation of Trasis AIO synthesiser** Prior to the production, the synthesiser underwent internal tests. After completion, the cassette was installed and evaluated using an automatic sequence to ensure that all connections had been secured in place and all components functioned properly.

**Preparation of dispensing** Dispensing was performed in a GMP Grade A hot cell equipped with fully automated open vial dose divider system from Von Gahlen. The incoming product line from the Trasis AIO was first rinsed with ethanol, followed by water for injection. A new dispensing set and appropriate sterile filters were then installed.

#### Preparation of reagent kit and cartridges

The reagent kit is composed of the following vials prepared in-house, in a GMP Grade C environment. All reagents were prepared on the day of the production, however the QMA eluent and precursor solution were made during cyclotron bombardment, as late as practically feasible before activity transfer.

1. **QMA eluent solution**. Tetraethylammonium bicarbonate (TEAB; 11.5 mg, 60.1 mmol) in acetonitrile/water (85/15 *v/v%*; 1.5 mL) for elution of [^18^F]fluoride from the QMA cartridge - attached to position 2 of the cassette.
2. **Precursor solution**. Tosylate-DPA 714 precursor (4 mg; 7.3 mmol) dissolved in anhydrous acetonitrile (1 mL) - attached to position 8 of the cassette.
3. **Quench solution**. 20% EtOH in water for injection - attached to position 10 of the cassette.
4. **Ethanol solution**. 70% EtOH in water for injection for elution of the tracer from the Sep-Pak Plus C18 cartridge - attached to position 12 of the cassette.
5. **0.9% NaCl solution**. Saline solution for injection - attached to position 16 of the cassette.
6. **HPLC product collection vial**. Vial prefilled with 14 mL water for injection for receipt of the HPLC product fraction - attached to the cassette via position 13 and the HPLC unit.

Prior to attachment to the cassette, the Sep-Pak C18 Plus cartridge was washed with absolute ethanol (5 mL), followed by water for injection (10 mL) and dried with air (5 mL). The preconditioned QMA cartridge supplied with the [^18^F]FDOPA cassette was used as such.

### GMP production of [^18^F]DPA-714

Cyclotron-produced [^18^O]H_2_O-bound [^18^F]fluoride (30-40 GBq) was first introduced into a Von Gahlen Activity Distribution System (ADS), where it was collected in a vial fixed inside an ISOMED 2010 dose calibrator from MED Nuclear Medizintechnik (Dresden, Germany), allowing for accurate and automatic activity measurements. Thereafter, it was distributed into the appropriate hot cell under a flow of helium (“Cyclotron” section). The difference between the accumulated activity (following target unloading) and the leftover activity (following receipt of the bulk [^18^F]fluoride volume in the designated Trasis AIO) was recorded as the starting activity. In the module, [^18^F]fluoride was trapped on a QMA cartridge, from which it was released with a solution of TEAB (40 mM; 0.75 mL) into a pre-heated reactor (60^∘^C). [^18^F]fluoride was dried by azeotropic distillation under stepwise heating. The reactor was cooled to 65^∘^C before addition of the tosylate DPA-714 precursor solution (4 mg) in anhydrous acetonitrile (1 mL). The mixture was heated to 90^∘^C for 10 min. After cooling to 60^∘^C, the reaction was quenched with 20% EtOH (1.5 mL) and injected on the HPLC unit for purification. The reactor and HPLC load lines were rinsed with the quench solution twice more (total volume of 8 mL) to ensure that the maximum amount of activity was recovered from the reactor. HPLC purification was performed on a Phenomenex Luna C18(2) column (100 Å 5 *μ*m; 250 ×10 mm) at room temperature, with 0.1 M ammonium acetate/ethanol (55/45 *v/v%*) as the mobile phase, running at 4 mL/min and a recording wavelength of 254 nm. The fraction containing [^18^F]DPA-714 (elution starting approximately 13-14 min post-injection) was collected into a sterile vial prefilled with water for injection (14 mL). The solution was withdrawn and passed through a Sep-Pak C18 Plus cartridge to remove the mobile phase. The cartridge was subsequently washed with 5 mL of saline, followed by elution of the trapped tracer with 70% ethanol (2 mL) and dilution with 0.9% NaCl solution for injection (8 mL).

Before arrival in a sterile bulk vial in the dispensing hot cell, the solution (approximately 10 mL) underwent sterile filtration on a 0.2 µm Pall Hydrophobic Stripe Membrane filter. Activity measurement was taken in an ionization chamber-based activity meter, situated in the dispensing hot cell (“??” section), followed by dilution with 0.9% NaCl to reach the required activity concentration of 400 ± 10% MBq/mL at EOS. The tracer solution was then mixed with nitrogen gas and dispensed using an automated system into sterile 10 mL injection vials, while being passed through a 0.22 µm sterilising Millex-GV PVDF filter (33 mm). The following vials were dispensed per production:

- **QC part 1 vial**. Initial QC was performed immediately after the production. It aimed to determine the product identity, chemical and radiochemical purity, pH, the presence of bacterial endotoxins and of residual tetraethylammonium (TEA) ions. Typically, 0.5-1 mL of the active product was filled.
- **QC part 2 vial**. Final QC was performed after decay (between 1-8 days post-production). It aimed to determine radionuclidic impurities, sterility and the presence of ethanol and residual solvents. Typically, 0.5-1 mL of the active product was filled.
- **Sterility vial**. The vial was sent for sterility testing to a third party. Results were made available a few weeks post-production. The vial typically contained 2 mL of the active product.
- **Reference and retention vial**. The vial was kept at Radboud Translational Medicine for reference purposes and unforeseen circumstances.
- **Customer vials**. These vials were filled according to customer requests, 400 MBq/mL at EOS – activity concentration at a required time.
- **Spare vial**. This vial was kept for emergency circumstances.
- **Stability vial**. This vial was filled only when stability studies were being undertaken.

### Validation of analytical procedures

#### HPLC for identification, chemical and radiochemical purity determination

Method validation was performed for HPLC identification and determination of chemical and radiochemical purity of the [^18^F]DPA-714 injection in compliance with ICH Guideline Q2(R1) Validation of Analytical Procedures and European Pharmacopoeia Chromatographic Separation Techniques 07/2016:20246.

The HPLC module consisted of the following Shimadzu (Kyoto, Japan) components: SPD-20A(V) UV-Vis detector coupled to a SIL-20AHT autosampler and injection unit, LC-20AT tandem plunger, CMB-20A light system controller, CTO-20AC column thermostat with a FCV-14AH6 6-way column switching valve and a DGU020A5R degassing unit. The system was coupled in series to an Berthold POMO PET radio flow detector (Bad Wildbach, Germany) for radioactivity measurements. The entire setup was controlled by DataApex Clarity software (Prague, Czech Republic).

Analysis was performed on a Waters XTerra Shield RP-18 column (125 Å 5 µm; 250 × 4.6 mm) at a flow rate of 1 mL/min using isocratic elution with 0.1 M ammonium acetate/acetonitrile (50/50 *v/v%*). The spectrum was recorded at 254 nm. Prior to [^18^F]DPA-714 injection, system suitability was validated by injection of a blank sample (water, 20 µL), followed by the DPA-714 reference solution (10 µg/mL, 20 µL) and an additional blank injection. Analysis of the radioactive sample was performed with the designated QC part 1 vial (“GMP production of [^18^F]DPA-714” section).

**Validation of chemical impurities (UV detector)** The following criteria were assessed during the validation: specificity, accuracy, linearity and precision. Range, detection and quantification limits were also determined. Linearity was essential for performing A_m_ calculations, using the slope and intercept of the line of best fit.

**Validation of radiochemical impurities (radiodetector)** The following criteria were assessed during the validation: specificity, accuracy, linearity and precision. Range, detection and quantification limits were also determined.

#### GC for residual solvent and ethanol content determination

Method validation was performed for gas chromatography (GC) identification and control of residual solvents in compliance with European Pharmacopoeia guidelines of Gas Chromatography 01/2008:20228, Residual Solvents 07/2016:50400, Identification and Control of Residual Solvents 07/2017:20424 and Chromatographic Separation Techniques 07/2016:20246, as well as the ICH guideline Q2(R1) Validation of Analytical Procedures. Ethanol was used as an excipient to enhance stability of the product and was not considered a residual solvent. The content of ethanol was determined to verify its compliance with a maximum of volumetric concentration of 10% *v/v%*.

The GC module consisted of the following Shimadzu components: GC-2010 PLUS unit equipped with an AOC-20 autoinjectior/autosampler and a hydrogen flame ionisation detector. The system was controlled by DataApex Clarity software. Analysis was performed on a Restek Rxi-624Sil MS column (30 m × 0.25 mm × 1.4 µm) with a gradient heating rate (0-2 min 40^∘^C, 2-7 min 80^∘^C, 7-11 min 160^∘^C) at a linear velocity of 15 cm/s with helium as carrier gas. Prior to [^18^F]DPA-714 injection, system suitability was validated by injection of a blank sample (water, 0.1 µL), followed by the reference solution containing 1-propanol, acetonitrile and ethanol (0.1 µL) and additional blank injection. Analysis of the batch sample was performed with a designated QC part 2 vial (“GMP production of [^18^F]DPA-714” section).

**Validation of GC analysis of residual solvents** The following criteria were assessed during the validation: specificity (acetonitrile, ethanol), linearity of ethanol and precision.

#### Thin layer chromatography spot test for determination of tetraethylammonium content

Despite the fact that TEAB is a commonly used reagent in [^18^F]fluorinations, to the best of our knowledge, no limit tests have been described in literature or the European Pharmacopoeia. The limit of TEA content was therefore chosen based on: 1) toxicity data for TEA obtained through animal studies and 2) the limit described for tetrabutylammonium (TBA), a longer alkyl chain derivative of TEA, used for [^18^F]PSMA-1007 radiosynthesis at Radboud Translational Medicine B.V. The lowest LD_50_ TEA value for intravenous injection (i.v.) was described for mice at 36 mg/kg by Pindell and co-workers (Pindell et al. 1961). Similar values were reported in the Merck Index (The Merck Index Online). For TBA, mouse median lethal dose (LD_50_) i.v. was estimated at 10 mg/kg, as reported by Meyer and co-workers (Meyer et al. 1995). Setting the limit of TEA content in radiopharmaceutical injections at the TBA value (0.26 mg/mL) was considered a generous and secure estimation.

Initial investigation of suitability of thin layer chromatography (TLC) spot test for detection of TEA in the [^18^F]DPA-714 injection was positive. Distinguishing of the TEA spot from other matrix components (ethanol, 0.9% sodium chloride) was done unambiguously using a TLC plate and iodine staining. The results are qualitative only, with no attempt to quantify the TEA content.

The following solutions were spotted adjacently on a TLC plate: matrix solution (10% ethanol in 0.9% NaCl), TEA reference solution (0.26 mg/mL) and undiluted [^18^F]DPA-714 solution. The plate was then placed in an iodine-saturated chamber at 50^∘^C for 2 min. Immediately, a picture of the plate was taken and assessed visually. TEA formed a distinct brown spot, while no spot were visible for the matrix solution. For compliance, intensity of the [^18^F]DPA-714 solution spot should be less than of the TEA reference solution. The amount of TEA for all validation productions was below the limit of detection.

**Validation of TLC spot test for TEA** The following criteria were assessed during the validation: specificity and detection limit.

#### Bacterial endotoxins test

Method validation was performed for determination of bacterial endotoxins in [^18^F]DPA-714 injection according to the guidelines of the European Pharmacopoeia on Bacterial Endotoxins 2.6.14.

Analysis was performed on a portable Endosafe PTS reader from Charles River Laboratories (Wilmington, USA). The following tests were performed during the validation: inhibition/enhancement screening and selection and verification of the final dilution factor.

### Quality control

Acceptance criteria were devised in consultation with general texts and monographs for established PET tracers, such as [^18^F]FDG and [^18^F]FDOPA of the current edition of the European Pharmacopoeia (Table 3). The TEA limit were formulated in-house (“Thin layer chromatography spot test for determination of tetraethylammonium content” section), while A_m_ limits were devised in consultation with clinicians involved with the planned [^18^F]DPA-714 study.

**Table 3:** Tests, methods and acceptance criteria for the quality control of [^18^F]DPA-714

Test	Method	Specifications at release
**At product release**		
Appearance	Visual inspection	Clear and colourless, free from visible particles
(Radio)chemical identity of [^18^F]DPA-714	HPLC (UV, gamma ray detectors)	[^18^F]DPA-714 peak in the radiochromatogram is consistent with the retention time of the reference standard to the nearest ±0.5 min, taking detector delay into account
Radionuclidic identity of fluorine-18	Gamma ray spectrometer	Energy of the principal gamma photon is 511 ±10 keV, possibility of sum peak at 1022 ±20 keV
Radionuclidic identity of fluorine-18 (half-life measurement)	Ionisation chamber	1.75-1.92 h
Radionuclidic purity of fluorine-18	Gamma ray spectometer	Fluorine-18 ≥99.9% of total activity
Radiochemical identity of [^18^F]DPA-714	HPLC (gamma ray detector)	Retention time of [^18^F]DPA-714 consistent with retention time of DPA-714 (±0.5 min)
Radiochemical purity of [^18^F]DPA-714	HPLC (gamma ray detector)	≥95% of total fluorine-18 activity
pH	pH strip	4.5-8.5
Chemical concentration of DPA-714	HPLC (UV detector)	≤0.01 mg/mL
Chemical concentration of any impurity above disregard limit	HPLC (UV detector)	≤0.01 mg/mL
Sum of DPA-714 and all impurities above disregard limit	HPLC (UV detector)	≤0.05 mg/mL
Molar activity	HPLC (UV detector)	≥100 GBq/µmol at EOS
Chemical concentration tetraethylammonium (TEA)	TLC spot test	≤0.26 mg/mL
Bacterial endotoxins	Chromogenic kinetic method	≤17.5 IU/mL
Sterile filter integrity	Bubble point test	≥2.8 bar
**After product decay**		
Radionuclidic impurities with half-life longer than 2 h	Gamma ray spectrometer	No signal higher than 5 times background noise
Residual solvents	Gas chromatography	Acetonitrile ≤0.41 mg/mL
Ethanol (excipient)	Gas chromatography	≤79 mg/mL
Sterility	Direct inoculation	Sterile

Quality control parameters that did no require method validation for this particular tracer were: pH, filter integrity test (FIT), radionuclidic identity, radionuclidic purity, sterility and environmental monitoring. The aforementioned were already validated for other radiopharmaceuticals produced on-site.

#### pH

Measurements were performed on a QUANTOFIX Relax reflexion photometer from Macherey-Nagel (Düren, Germany) using their pH-Fix 2-9 indicator strips.

#### Filter integrity test

Integrity of the final sterilising filter was assessed by a bubble point test. This was performed automatically via an in-line FIT system of the Von Gahlen open vial dose divider used for the dispensing process.

#### Radionuclidic identity and purity

**Identity** Radionuclidic identity was determined from half-life measurements, on the ISOMED 2010 dose calibrator with ISOMED software control, after dispensing.

**Purity** Radionuclidic purity was determined using an Osprey Multi-channel Analyser from Canberra Industries (Meriden, United States) using the resulting gamma ray spectrum.

#### Sterilty

Sterility of the final product was determined by direct inoculation in soybean casein digest and fluid thioglycollate media. This was performed by a third party, Eurofins Bactimm (Nijmegen, Netherlands).

#### Environmental monitoring

Environmental monitoring was performed using contact and settle Trypticase Soy Agar plates with lecithin and polysorbate 80 isolator pack, acquired from Eurofins Bactimm, placed within 30 cm of critical operations in all GMP Grade A manipulation areas concerned - dispensing hot cells and airlocks. The plates were sent to Eurofins Bactimm for analysis.

### Stability studies

For stability studies, three [^18^F]DPA-714 productions were performed. QC analysis involving visual inspection, HPLC and pH measurements were performed at release and 6 hours post-EOS. The sample was stored in a designated vial, in an inverted position, to mimic vial flipping during transport.

## Results

### Optimisation of radiochemistry

In optimisation studies, radiofluorination of tosylate DPA-714 was investigated with different PTC/base systems in acetonitrile. The subset included the most commonly used PTC, Kryptofix-222, in conjunction with potassium bases - carbonate and bicarbonate. TEAB was also tested. The procedure was very similar for each experiment. The reaction mixture was heated to 90^∘^C for a fixed time interval of 10 min. Table 1 summarises the results. Differences in elution volumes are attributed to the efficiency of [^18^F]fluoride release from the Sep-Pak QMA cartridge, measured as trapped activity with a dose calibrator.

A similar reaction profile was obtained for all three runs, with [^18^F]DPA-714 as the main reaction product, with unreacted [^18^F]fluoride as the only other radioimpurity. [^18^F]Fluorination proceeded most efficiently in the presence of TEAB. Almost quantitative RCY, 94%, was obtained, as estimated by (radio)HPLC. With optimised conditions in hand, preparation of the first automated production on a Trasis AIO followed thereafter.

### Automated production

Suitable semi-preparative HPLC purification conditions were also identified (described in full detail in “GMP production of [^18^F]DPA-714” section).

**Fig. 3:**
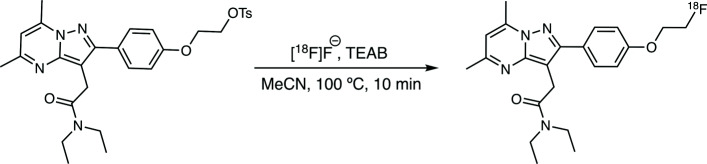
Radiosynthesis of [^18^F]DPA-714 using optimised conditions. OTs = tosylate

**Fig. 4:**
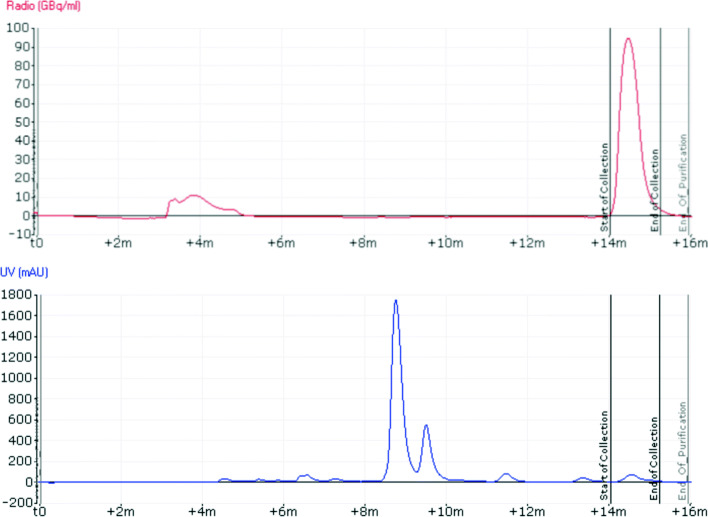
HPLC purification chromatogram of a [^18^F]DPA-714 production on the Trasis. Upper trace: Radioactivity. Note: the scale is not calibrated, so radioactivity values are not accurate. Lower trace: UV measurement, *λ* = 254 nm

Several automated test runs were performed based on the most appropriate conditions identified during the manual labelling stage (“Optimisation of radiochemistry” section). Cosmetic changes had to be made to the process to accommodate for intrinsic differences between manual and automated radiolabelling. In this case, for best results, reaction temperature had to be altered (from 90^∘^C hotplate setting to 100^∘^C on Trasis AIO) to compensate for commonly encountered differences in the heating rate of various sources. In addition, it was important to change the volume of the QMA eluent (0.6 mL for manual labelling versus 0.8 mL for Trasis AIO), based on unavoidable liquid losses in long transfer tubings of automated synthesisers. The most suitable conditions for automated labelling involved elution of the [^18^F]fluoride off the QMA cartridge with 0.8 mL of TEAB solution, followed by stepwise azeotropic distillation (with no extra acetonitrile added) and cooling to 65^∘^C before addition of the precursor (4 mg) dissolved in acetonitrile (1 mL). The reaction was carried out at 100^∘^C for 10 min (Fig. 3). The quench solution contains 20% ethanol, for added stability and better tracer recovery from the reactor and tubing. Following HPLC purification, the tracer was trapped on an solid-phase extraction (SPE) cartridge, from which it was released with 70% ethanol, an amount sufficient to elute the compound from the sorbent quantitatively, as well as tolerated by the first sterilising filter. The semi-preparative HPLC chromatogram of the purification is shown in Fig. 4. Similarly to manual labelling experiments, [^18^F]DPA-714 was the major radioactive product. Unreacted [^18^F]fluoride was easily separated by HPLC.

[^18^F]DPA-714 production was validated for routine clinical use according to GMP specifications. Three equivalent validation productions were performed. QC analysis including stability assessment was completed. RCYs (dc.) for the respective productions were 62, 70 and 71%. The total synthesis time, from the receipt of activity in the Trasis AIO to arrival of the formulated product in the dispensing unit, was approximately 60 min. Following arrival in the dispensing module, the mother solution (9 ± 1 mL) was diluted to reach the required radioactivity concentration of 400 ± 10% at EOS in the bulk vial. Reaction parameters, yields and concentrations for the validation process are presented in Table 4.

**Table 4:** Batch information: radioactivity parameters of the automated process

Batch information	Batch 1	Batch 2	Batch 3
Starting activity [GBq]	41.82	42.38	34.49
RCY (ndc.) [%]	42	48	47
RCY (dc.) [%]	62	71	70
Activity in bulk vial (post-reaction) [GBq]	17.37	20.31	16.12
Volume in bulk vial (pre-dilution) [mL]	9.83	10.43	10.11
Volume in bulk (post-dilution) [mL]^a^	34.39	31.32	28.84
Activity concentration in bulk vial [MBq/mL]	505	648	559
Synthesis duration [min]^b^	63	62	64

^a^Dilution with sterile 0.9% NaCl

^b^From arrival of activity in AIO to measurement of activity in dispensing unit (pre-dilution)

### Validation of analytical procedures

#### HPLC (UV detector)

**Fig. 5:**
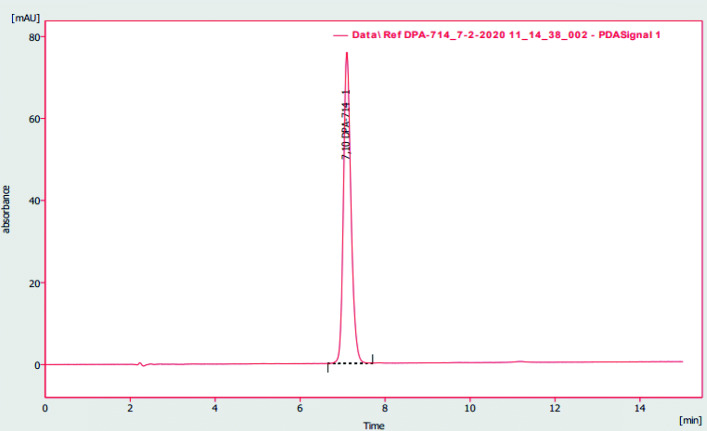
Typical HPLC chromatogram (*λ* = 254 nm) obtained with a DPA-714 reference standard (10 µg/mL)

The HPLC method described in “HPLC for identification, chemical and radiochemical purity determination” section allowed for unequivocal identification of DPA-714 from other components of the matrix. Retention time of the compound was 7.1 min (Fig. 5). The tailing factor of the corresponding peak was 1.238, well in the specified range of 0.8-1.5. Resolution between DPA-714 and any other impurity was 14.271, in line with the acceptance criterion. Accuracy, expressed as percentage recovery, complied with the limit of 90-110% for the chosen concentrations. Precision was assessed through the coefficient of variation (CV) of the area under the peak, which did not exceed the specified 5% for three injections at the same concentration (2.07, 1.72 and 0.08). Linearity and range were evaluated by constructing a calibration curve. The correlation coefficient, R, was 0.9999, while the range was found to be 0.1-12 µg/mL. The limit of detection (LOD) was established at 0.03 µg/mL and the limit of quantification (LOQ) at 0.09 µg/mL. The results confirmed suitability of the chosen analytical HPLC method for the quality control of a [^18^F]DPA-714 solution for injection.

#### HPLC (radiodetector)

**Fig. 6:**
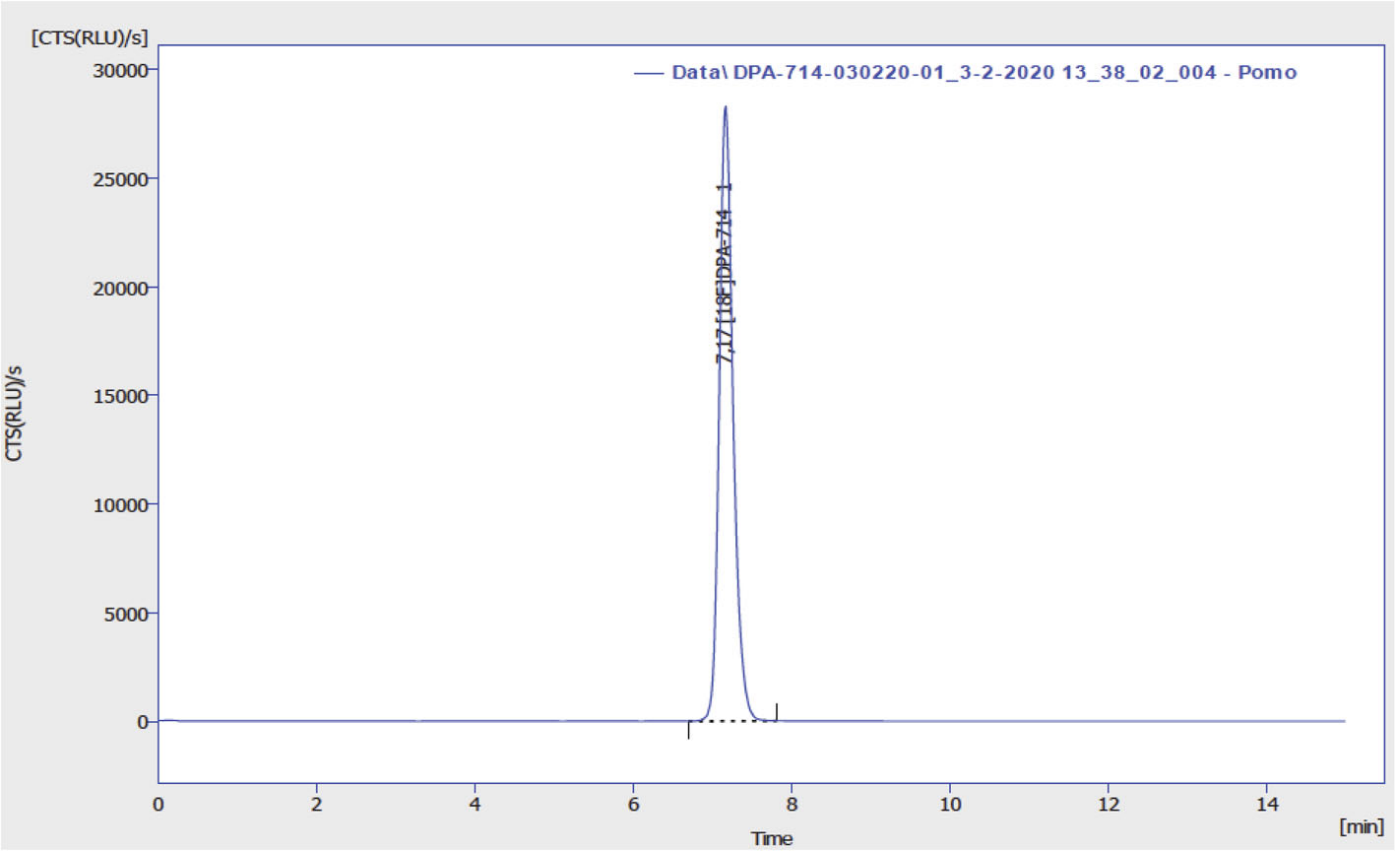
Typical HPLC radiochromatogram obtained with [^18^F]DPA-714 solution. RCP = 100%

The HPLC method described in “HPLC for identification, chemical and radiochemical purity determination” section allowed for unequivocal identification of [^18^F]DPA-714 from any other [^18^F]fluorinated compound in the solution. Retention time of [^18^F]DPA-714 was 7.17 min (Fig. 6). The tailing factor of the corresponding peak was 1.227. Retention time of [^18^F]DPA-714 corresponded to the retention time of the DPA-714 reference standard (±0.5 min), taking the sequential use of detectors into consideration. No other peaks were identified. Accuracy, expressed as percentage recovery, complied with the limit of 90-110% for the chosen activity concentrations of 250, 500 and 750 MBq/mL. Precision was assessed through the CV of the area under the peak, which did not exceed the specified 5% for three injections at the same activity concentration (0.51, 0.90 and 1.01). Linearity and range were evaluated by constructing a calibration curve. R was 0.9999, while the range of the method was found to be 0.7-750 MBq/mL. LOD was established at 0.2 MBq/mL and LOQ at 0.7 MBq/mL. The presented results proved suitability of the chosen analytical HPLC method for the quality control of a [^18^F]DPA-714 solution for injection.

#### GC

The GC method described in “GC for residual solvent and ethanol content determination” section allowed for accurate and specific measurement of acetonitrile (residual solvent) and ethanol (excipient) in the presence of other components of the sample matrix of a sterile [^18^F]DPA-714 solution for injection. The symmetry factor of the solvents was, according to the acceptance criterion, smaller than 2: 0.995 for ethanol, 1.103 for acetonitrile and 1.181 for 1-propanol. Resolution between acetonitrile/1-propanol and ethanol was 10.479 and 10.765, respectively. Linearity and range for ethanol were evaluated by constructing a calibration curve. R was found to be 0.9994 and the concentration of ethanol linear within the range of 1-100 mg/mL. System repeatability for acetonitrile and ethanol was assessed through the relative standard deviation (RSD), which did not exceed the specified 15% - it was 0.55 for the ethanol/1-propanol and 0.90 for acetonitrile/1-propanol. The presented results prove suitability of the chosen GC method for the determination of acetonitrile and ethanol content in a [^18^F]DPA-714 solution for injection.

#### Bacterial endotoxins test

Dilutions of 10 and 100 complied with the specified spike recovery between 50 and 200%, however the former exhibited better accuracy. The results proved suitability of the test for the determination of bacterial endotoxins in a [^18^F]DPA-714 solution for injection.

#### Tetraethylammonium

LOD was qualitatively (visually) established as 0.01 mg/mL of TEA ions. No spots corresponding to any component of the matrix or the [^18^F]DPA-714 solution were visualised using the method described in “Thin layer chromatography spot test for determination of tetraethylammonium content” section, apart from TEA itself. The presented results prove suitability of the method for TEA determination in a [^18^F]DPA-714 solution for injection.

### Quality control (validation productions)

[^18^F]DPA-714 was synthesised in excellent chemical and radiochemical purity (RCP), the latter reaching 100% (n=3), as determined by (radio)HPLC. Retention time of the radioactive peak was consistent with that of the DPA-714 reference standard to the nearest 0.5 min. Good A_m_ values were obtained: 173, 211 and 117 GBq/µmol. No TEA residues were revealed in any of the batches. Acetonitrile and ethanol content was well below their corresponding limits. The synthetic process has an extremely low bioburden associated. The final sterile filter remained intact, leading to a sterile solution for injection. Table 5 presents all quality control results obtained for three validation batches. Detailed explanation of acceptance criteria is outlined in Table 3. All parameters were in compliance with the specifications.

**Table 5:** Quality control results at product release for three validation batches of [^18^F]DPA-714. Detailed explanation of acceptance specifications can be found in Table 3

Test	Batch 1	Batch 2	Batch 3	Complies/Does not comply
**At product release**				
Appearance	Clear, colourless	Clear, colourless	Clear, colourless	Complies
Radionuclidic identity of fluorine-18 [keV]	511 ±10	511 ±10	511 ±10	Complies
Radionuclidic identity of fluorine-18 [h]	1.82	1.82	1.81	Complies
Radionuclidic purity of fluorine-18 [%]	≥99	≥99	≥99	Complies
Radiochemical identity of [^18^F]DPA-714 [min]	0.36	0.32	0.03	Complies
Radiochemical purity of [^18^F]DPA-714 [%]	100	100	100	Complies
pH	6.8	6.6	6.8	Complies
Chemical concentration of DPA-714 [mg/mL]	≤0.01	≤0.01	≤0.01	Complies
Chemical concentration of any impurity above disregard limit [mg/mL]	≤0.01	≤0.01	≤0.01	Complies
Sum of DPA-714 and all impurities above disregard limit [mg/mL]	≤0.05	≤0.05	≤0.05	Complies
Molar activity [GBq/µmol]	173	211	117	Complies
Chemical concentration TEA [mg/mL]	<0.26	<0.26	<0.26	Complies
Bacterial endotoxins [IU/mL]	<0.5	<0.5	<0.5	Complies
Sterile filter integrity [bar]	3.48	3.38	3.38	Complies
**After product release**				
Radionuclidic impurities with a half-life longer than 2 h	None	None	None	Complies
Acetonitrile [mg/mL]	0.02	0.03	0.02	Complies
Ethanol [mg/mL]	34	42	40	Complies
Sterility	Sterile	Sterile	Sterile	Complies
Bioburden [CFU]^a^	<1	<1	<1	Complies

^a^Only tested during the validation phase. Not part of regular QC

### Stability studies

The three batches were tested for stability during a 6-hour post-EOS interval, allowing for completion of the initial QC, transport to the designated centre, with potential delays at any stage prior to patient injection. During the study, the volume of product and position with respect to the vial septum were altered. For each batch, two vials containing 0.5 and 10 mL of the product were dispensed and stored at 15−25^∘^C in the inverted position to test chemical integrity of the formulation upon uninterrupted interaction with the septum, acting as the worst case scenario for storage. After 6 h, the samples were assessed visually for colour and transparency changes, pH, and chemical/radiochemical purity using (radio)HPLC, variations of which could be indicative of limited capacity of the excipient (ethanol) as a stabiliser and/or a chemical reaction between the components of the formulation and the septum, resulting in undesired peaks on chromatograms and/or significant colour and pH changes. Satisfyingly, in all instances, the formulation were stable for a minimum of 6 hours post-EOS. The product retained 100% RCP, with no changes to the chemical composition, as judged by (radio)HPLC (using the method outlined in “HPLC for identification, chemical and radiochemical purity determination” section). The pH remained stable with respect to measurements at EOS. The data is shown in Table 6.

**Table 6:** Results of the stability study performed 6 hours post-EOS for three validation batches of [^18^F]DPA-714. Detailed explanation of acceptance specifications can be found in Table 3

Test	Batch 1	Batch 2	Batch 3
	Vial 0.5 mL Vial 10 mL	Vial 0.5 mL Vial 10 mL	Vial 0.5 mL Vial 10 mL
Appearance	Clear, colourless	Clear, colourless	Clear, colourless
pH	6.9 6.8	6.5 6.9	6.4 6.5
Radiochemical identity of [^18^F]DPA-714 [min]	0.34 0.03	0.37 0.35	0.03 0.34
Radiochemical purity of [^18^F]DPA-714 [%]	100	99.7 100	100
Chemical concentration of DPA-714 [mg/mL]	≤0.01	≤0.01	≤0.01
Chemical concentration of any impurity above disregard limit [mg/mL]	≤0.01	≤0.01	≤0.01
Sum of DPA-714 and all impurities above disregard limit [mg/mL]	≤0.05	≤0.05	≤0.05

### Routine clinical productions

Until the time of submission of this publication, three GMP-complaiant productions were performed for patient studies. RCYs were 55, 68 and 55%, with molar activities of 350, 232 and 227 GBq/µmol, respectively. The productions all conformed to the specification criteria at and post-release.

## Discussion

Following a short series of manual labelling experiments, highly promising labelling conditions were identified, eradicating the need for further investigation. Radiolabelling follows nucleophilic aliphatic substitution (S_N_2) on a tosylate leaving group (Figure 3). The reaction proceeded cleanly with no distinct radioimpurities. The unreacted [^18^F]fluoride was easily separated during semi-preparative HPLC purification. Interestingly, when a test run was performed without HPLC purification and relying solely on solid-phase extraction, co-eluting impurities were observed which significantly lowered RCP to <95%. Unsurprisingly, RCY obtained during manual labelling could not be reproduced on an automated system due to limited control over some variables (such as mixing and rinsing) and inherent losses of solutions/reaction mixture during numerous transfer stages.

Automation on the Trasis AIO module greatly reduces the level of complexity that characterises, still commonly used, non-cassette based synthesisers. The use of single-use GMP-grade cassettes, tubing and reagent vial not only reduces the risk of cross-contamination and reduces cleaning but very importantly, lowers bioburden of the process. The programmed sequence is robust and based on the presented results, the level of batch-to-batch variability is very low. This greatly simplifies training of production personnel. The sequence could naturally be automated to greater extent, however it was deemed less beneficial from the radiosynthetic aspect. For example, automatic HPLC collection, especially when a fixed duration is set, may jeopardise the entire production, if yields are lower than expected. Nevertheless, our proposed strategy could easily be translated to any other automated synthesiser.

Following successful validation procedure, including three fully automated productions and aseptic dispensing, the described GMP-compliant [^18^F]DPA-714 radiosynthesis delivered the tracer in, to the best of our knowledge, the highest RCY reported in literature and the first automated protocol designed on a Trasis AIO module. Several patients can be scanned with a single batch of the tracer, following short cyclotron irradiation. Our method offers a clear advantage over other published methods - significantly higher RCYs, 55-71% (n=6) vs 43-50%, both dc., obtained with the same precursor loading and with similar starting activities. The main difference between the approach of Kuhnast et al. is in the PTC. TEAB could therefore be a better enhancer of [^18^F]fluoride nucleophilicity than Kryptofix-222, perhaps due to better stabilisation of the precursor in the reaction. Fookes et al. reached very high [^18^F]DPA-714 of 55-85% (dc.) for a smaller precursor loading of 2 mg (Fookes et al. 2008). The difference could be ascribed to the purification and formulation protocol - following HPLC purification, the solvents were removed in vacuo, rather than by SPE, hence reducing the number of transfers and losses due to tracer sticking to tubings, etc. Given the scale of clinial production, this would be neither practical nor safe, with a low degree of batch-to-batch reproducibility.

Quality control tests performed on all the batches were fully compliant with in-house and European Pharmacopoeia specifications for the synthesis of ^18^F-based tracers for routine clinical use. The product remains intact (RCP of 100%) at high activity concentrations for a minimum of 6 hours, without the need of extra stabilisers, such as sodium ascorbate.

Radboudumc is currently involved with a clinical study employing [^18^F]DPA-714 as a neuroinflammation biomarker, produced using our GMP-compliant method, on a regular basis.

## Conclusion

[^18^F]DPA-714 was synthesised in 55-71% RCY (n=6) in a nucleophilic aliphatic substitution on a tosylate leaving group with [^18^F]fluoride mediated by TEAB in acetonitrile. The method represents a significant improvement to the existing protocols, which use a similar or increased precursor loading. One of its assets, apart from robustness and reliability, is, undeniably, the use of an automated module with a disposable cassette, easily implementable in a modern radiopharmacy laboratory. The tracer can be produced in sufficient amounts, following short bombardment, to allow for shipment to more remote centres with no impact on tracer stability. The process and quality control comply with GMP standards, rendering it suitable for routine human use.

## Data Availability

Data can be obtained upon request. Declarations

## Abbreviations

AIO: AllinOne
A_m_: Molar activity
AD: Alzheimer’s disease
CFU: Colony-forming unit
CV: Coefficient of variation
dc.: Decay-corrected
EOS: End of synthesis
EtOH: Ethanol
[^18^F]FDG: 2-Deoxy-2-[^18^F]fluoroglucose
FIT: Filter integrity test
GBq: Gigabecquerel
GC: Gas chromatography
GMP: Good manufacturing practice
h: Hour
HPLC: High performance liquid chromatography
IU: International unit
i.v.: Intravenous injection
K_222_: Kryptofix-222
LD_50_: Median lethal dose
LOD: Limit of detection
LOQ: Limit of quantification
MBq: Megabequerel
MS: Multiple sclerosis
ndc.: Non-decay-corrected
OD: Outside diameter
PBR: Peripheral benzodiazepine receptor
PET: Positron emission tomography
PTFE: Polytetrafluoroethylene
PVDF: Polyvinylidene fluoride
RCP: Radiochemical purity
RCY: Radiochemical yield
PTC: Phase-transfer catalyst
QC: Quality control
QMA: Quaternary methylammonium
R: Correlation coefficient
RSD: Relative standard deviation
SPE: Solid-phase extraction
TBA: Tetrabutylammonium
TEA: Tetraethylammonium
TEAB: Tetraethylammonium bicarbonate
TLC: Thin layer chromatography
TSPO: Translocator protein
